# Age-related changes in connected speech production: evidence from eye-tracking in the culturally adapted picture description task

**DOI:** 10.3389/fpsyg.2024.1334788

**Published:** 2024-08-22

**Authors:** Hyeri Lee, Yoomi Choi, Jee Eun Sung

**Affiliations:** ^1^Department of Communication Disorders, Ewha Womans University, Seoul, Republic of Korea; ^2^Department of Media Interaction Design, Ewha Womans University, Seoul, Republic of Korea

**Keywords:** age-related changes, connected speech, picture description tasks, eye tracking, cultural adaptation

## Abstract

**Purpose:**

Age-related changes in connected speech production remain a subject of debate, yielding inconsistent findings across various tasks and measures. This study aimed to investigate the effects of aging on picture description tasks using two types of pictures: a standardized picture (the Beach picture) and a culturally and linguistically modified picture tailored for Korean speakers (the Han River picture).

**Method:**

Twenty-four young adults and 22 older adults participated in two picture description tasks while their eye movements were recorded. Word-level linguistic variables were used to assess informativeness (Correct Information Units per minute) and productivity (noun and verb counts per utterance) of connected speech production. Eye-movement measures were employed to evaluate real-time cognitive processing associated with planning connected speech (pre-speech fixation counts and durations; eye fixations before the speech onset of each utterance).

**Results and conclusions:**

The findings revealed age-related declines in linguistic measures, with older adults exhibiting decreased CIUs per minute and smaller counts of nouns and verbs per utterance. Age-related changes in eye movement measures were evident in that older adults displayed longer pre-speech fixation durations. Unlike younger adults, older adults exhibited higher pre-speech fixation counts on the Han River picture compared to the Beach picture, suggesting cognitive challenges in performing the task that requires producing more words and detailed descriptions. These results suggest that aging is associated with reduced informativeness and productivity of connected speech, as well as a decline in cognitive processing efficiency.

## 1 Introduction

The aging population has been increasing globally at an unprecedented rate. A recent report from the World Health Organization projected that one in six people worldwide will be aged 60 years or older by 2030. Among the significant concerns associated with aging are the risks of neurodegenerative conditions, such as dementia or mild cognitive impairment, which have posed substantial socioeconomic burdens on family caregivers and governments. Early detection of cognitive-linguistic changes associated with aging could be used as a guide for normal aging and clinical populations in the prevention and treatment of neurodegenerative conditions.

Recent research has aimed to capture subtle alterations in the cognitive-linguistic abilities of older adults through language production tasks. Some studies sought to demonstrate that early symptoms can be discerned by analyzing connected speech samples obtained from tasks such as picture descriptions, interviews, or storytelling tasks (cf. Mueller et al., 2016; Filiou et al., 2019). Connected speech refers to spontaneously spoken discourse with minimal speaker monitoring, representing the most systematic form of language production (Ash and Grossman, 2015). Connected speech production imposes various cognitive and linguistic demands on the speakers, including processing speed, working memory, attention, semantic storage and retrieval, syntactic formulation, and alignment with a communication goal or topic to create a coherent discourse (cf. Griffin and Spieler, 2006; Collette et al., 2009; Mueller et al., 2018). Given its complexity and cognitive demands, examining connected speech production may serve as a valuable method to detect age-related changes in cognitive-linguistic domains among aging populations. Moreover, it offers an opportunity to identify changes in language production that are more akin to real-life communications than to the language elicited by the tasks of single-word naming or verbal fluency.

The primary objective of this study is to explore the linguistic and cognitive attributes associated with connected speech production, aiming to discern age-related change among young-old adults (typically defined as individuals in late middle age to early old age). Existing evidence from language production studies suggests that the aging process manifests from late midlife to young-old age. Schmitter-Edgecombe et al. (2000) reported that the young-old group (aged 58–74 years) exhibited a higher proportion of word-finding errors compared to younger adults (aged 18–22 years). Also, Verhaegen and Poncelet (2013) reported slowed processing in word retrieval from the age of 50. While individuals in their 50s and younger adults (aged 25–35) exhibited no difference in naming accuracy, those in their 50s showed significantly longer naming latencies. A longitudinal study by Connor et al. (2004) documented a gradual decline in word retrieval abilities over time among participants aged 30 to 87, indicating that the participants in the late midlife and young-old age were experiencing a decline in naming abilities.

The extent to which aging affects connected speech production, particularly in terms of word retrieval, has been a matter of debate. The variability in research findings is partly attributed to two factors: the diverse tasks and various linguistic outcome measures employed in previous studies (Kavé and Goral, 2016). To begin with, the task paradigms have ranged from picture description to story narration and to conversations or interviews (Saffran et al., 1989; Schmitter-Edgecombe et al., 2000; Kemper and Sumner, 2001; Boschi et al., 2017). Older adults tended to produce more words (James et al., 1998) and retrieve a greater variety of words (Kemper and Sumner, 2001) in spontaneous conversations or interviews. In contrast, older adults were less talkative (James et al., 1998) and showed a more restricted selection of words (Capilouto et al., 2015) in structured tasks such as picture descriptions.

One of the most frequently employed methods to elicit connected speech samples is the picture description task. In the task, participants are presented with a picture depicting characters engaged in familiar activities and instructed to describe it (Brookshire and Mcneil, 2014). It creates a constrained environment for identifying intended words and assessing the success of word retrieval, enabling easy cross-group comparisons (Chenery and Murdoch, 1994). Picture descriptions are widely adopted and proven to be invaluable across diverse populations, ranging from healthy older adults to individuals with neurodegenerative diseases and aphasia (Ash and Grossman, 2015; Drummond et al., 2015; Vandenborre et al., 2017; Hameister and Nickels, 2018; Mueller et al., 2018; Boucher et al., 2019; Richardson and Dalton, 2019). Moreover, the task enables researchers to examine real-time measures of eye movements as the speakers are looking at the picture while performing the task. Eye movements have been suggested to provide valuable insights into the cognitive processes underlying language production. Notably, prolonged and frequent eye fixations are indicative of heightened cognitive processing demands (Rayner, 1998; Schotter, 2011).

In adopting picture description tasks, studies have used various types of pictures, including single pictures, sequential pictures, or story pictures (cf. Mueller et al., 2018). Recently, researchers have emphasized cultural and linguistic factors in the pictures that may significantly influence speakers’ interpretation and description of them. Some studies attempted to enhance linguistic and cultural sensitivity in pictures. The ‘Cookie Theft’ picture from the Boston Diagnostic Aphasia Examination (BDAE; Goodglass and Kaplan, 1972), which was standardized and is the most commonly used picture stimuli in the field of communication disorders, has been critiqued for containing gender, racial, and socioeconomic stereotypes (Steinberg et al., 2022). Thus, there have been attempts to address these limitations by modifying the background scene and characters and adding more objects and actions to it (Berube et al., 2019). Additionally, some studies have examined the influence of ethnicity on descriptions of the Cookie Theft picture by comparing the connected speech of Black/African Americans and non-Hispanic whites (Evans et al., 2022). Another study compared Turkish speakers’ descriptions of the Cookie Theft picture to a picture tailored for Turkish speakers and found the tailored one elicited more distinctive morphosyntactic features unique to the Turkish language (Seçkin and Savaş, 2023). These studies demonstrate the importance of using pictures that resonated with participants’ linguistic, cultural, and social backgrounds to effectively capture linguistic characteristics and semantic knowledge in connected speech samples (Mueller et al., 2018). Therefore, picture stimuli tailored for a specific language would produce a more accurate assessment of the cultural and linguistic differences of diverse populations.

In South Korea, the Beach picture from the Paradise-Korean version of the Western Aphasia Battery-Revised (PK-WAB-R; Kim and Na, 2012) has been the standardized picture most commonly used in picture description tasks, both in research and clinical settings. It has also been extensively utilized in various populations including healthy young and older adults (Kwon et al., 1998; Lee and Kim, 2001; Jeon and Kim, 2015; Kim et al., 2015; Choi, 2020), individuals with dementia (Kim et al., 2006; Ha et al., 2009), and those with aphasia (Kim et al., 1998; Lee et al., 2009; Kim and Sung, 2022), to name a few. However, the Beach picture exhibits certain inherent linguistic and cultural issues. The objects and actions depicted in the picture may be less familiar to Korean speakers. This is because the Beach picture was adopted from the Western Aphasia Battery (Kertesz, 2007), with its fundamental scenery remaining unchanged. Furthermore, the picture portrays limited events that may not sufficiently elicit action-related information or specific lexical items, such as verbs. The presence of only six active agents (see ‘Materials and methods’ section for further detailed descriptions), contributes to this limitation. This presents a crucial concern given that Korean is characterized as a pro-drop and verb-salient language, which allows the omission of verb arguments (e.g., subject or object, which are nouns) from a sentence when their identity can be inferred from context (Kwon et al., 2006; Jung and Lee, 2018). This verb saliency in Korean has been observed in previous studies on connected speech, where Korean speakers with aphasia demonstrated a greater number of verbs compared to that of English speakers with aphasia for the same picture description tasks (Sung et al., 2016).

With the limitations of the Beach picture in consideration, Sung and colleagues (Jeong et al., 2023) introduced a new picture called the ‘Han River’ picture (Patent no. D2022-0004KR), which was specifically designed for Korean speakers. They took the verb saliency of the Korean language as a key variable and included diverse action drawings in the picture scene in order to elicit more verbs (e.g., daily activities seen on the river banks). Furthermore, cultural familiarity was enhanced by depicting the background scene as the Han River and including daily activities happening there. The Han River holds a symbolic significance in Korean cultural identity as the river passes through the center of Seoul. It has served as a social and economic hub for residents and visitors alike and represents a familiar space intertwined with the daily lives, aspirations, and emotions of Korean people (Baik and Kim, 2012). Along the river bank parks, people gather to exercise, have picnics, and take leisurely walks while enjoying the scenic river.

One of the objectives of this study was to investigate how different picture types influence age-related differences in the task of describing picture scenes. We are particularly interested in whether the Han River picture would evoke a greater number of information units and action-related words, particularly verbs for Korean speakers. In Korean, verbs are hypothesized to impose a greater cognitive load due to their complex grammatical structure. Korean sentences follow an “SOV” (Subject-Object-Verb) structure, where verbs typically appear at the end of the sentence. Thus, activation of semantic and syntactic elements preceding the verb would be necessary before its production. Previous studies with the tasks of single-word production have already shown the age-related difficulties in producing heavy verbs compared to light verbs in generative naming tasks (Choi et al., 2021), as well as challenges in generating verbs compared to nouns in verbal fluency tasks (Östberg et al., 2005; Stokholm et al., 2013; Clark et al., 2014). However, to our knowledge, no studies have specifically investigated age-related changes in eliciting verbs within the context of connected speech. By utilizing the Han River picture, which was designed to draw out many verbs, our study aimed to investigate age-related changes in verb production abilities in connected speech.

Turning to the second factor, outcome measures in previous studies have also shown considerable variation. Thus far, the examination of outcome measures in language studies involved two analyses: (1) linguistic variable analysis and (2) real-time variable analysis. First, different linguistic variables have been used in connected speech analysis, which includes productivity (e.g., the number of words produced) and communication efficiency (e.g., correct words per unit of time). These measures have yielded mixed results, reporting both age-related declines and no significant changes. Some studies have reported significant age-related decreases in the number of produced words (Heller and Dobbs, 1993; Kemper and Sumner, 2001; Cho et al., 2021), while others have found no change with age (Cooper, 1990; Castro and James, 2013; Capilouto et al., 2015). Additionally, some studies have found fewer propositions relative to the total number of words produced (MacKenzie, 2000), while others have reported no decline in measures such as the number of propositions per minute (Cooper, 1990), the number of words needed to convey an information unit (Boucher et al., 2019), or the number of Correct Information Units per minute (Capilouto et al., 2005).

Among those linguistic measures, Correct Information Units (CIUs) serve as one of the most frequently used linguistic measures for capturing semantic deficits across languages. Originally developed by Nicholas and Brookshire (1993), CIUs represent words that are both (1) intelligible in context and (2) accurate, relevant, and informative about the picture or topic of connected speech. To count CIUs, intelligible words are first collected, excluding unintelligible words or non-word fillers. From those intelligible words, CIUs are determined according to the specific criteria; importantly, they do not have to be used in a grammatically correct manner, but words that incorrectly describe the picture or attempts to correct sound errors are excluded (see Nicholas and Brookshire, 1993, for further details).

The current study investigated age-related differences in word retrieval ability in the context of connected speech production by utilizing CIUs per minute and counting the number of nouns and verbs produced. These single-word measures were employed since word retrieval was the domain of interest. The CIUs per minute represented informativeness, and the number of nouns and verbs showed productivity in connected speech samples. Nouns and verbs were chosen as dependent measures because they constitute the core lexicon in Korean, making them representative indicators of lexico-semantic retrieval abilities and core words in constructing sentences.

Secondly, real-time measures, such as eye movements, have been employed in language production tasks. Eye-tracking can provide a direct measure of visual attention and cognitive effort during picture description tasks, which is an aspect not fully captured by analyses of spoken data after task completion (Rayner, 1998; Henderson and Ferreira, 2004; Schotter, 2011; Conklin et al., 2018). Given the complex interplay between cognitive and linguistic processes in picture description tasks, the cognitive-linguistic challenges faced by older adults may result in distinct eye movement patterns compared to the ones from younger adults. During these tasks, speakers must visually process a scene, identify key elements, retrieve and organize words, and make inferences about relationships among the depicted elements (Cummings, 2019). Age-related changes in cognitive processes, such as declines in working memory capacity and attentional control (Salthouse, 1990; Zacks and Hasher, 1994), may affect the process of generating verbal descriptions. For instance, decreased working memory resources may hinder the maintenance of active lexical representations for production and the tracking of information already produced in preceding utterances (Martin and Slevc, 2012; Marini, 2023).

Researchers have temporally synchronized language and eye movement data to investigate cognitive processing during a specific period of time. Eye movements have been scrutinized during the planning stage of language production when speakers were to comprehend events, assign roles to subjects, and possibly select verbs (Meyer and Dobel, 2003). While speakers selected words and assembled phonemes to name specific objects, eyes often fixated on them (Meyer et al., 1998; Griffin, 2004). Many studies consistently reported a close temporal relationship between eye movements and language production. The time interval between initiating eye gaze on an object and uttering its name typically remains consistent across studies, which lasts approximately a second. Even if speakers had previously fixated on an object, they often redirect their gaze back to it for about a second before naming it (Griffin and Bock, 2000). Additionally, the duration of gaze on an object before its naming reflects one’s cognitive efforts that are required to name it (Griffin, 2004). For example, individuals tend to spend more time observing objects before verbally describing them rather than simply scanning them to form a general impression (Griffin and Bock, 2000). These findings collectively suggested a deliberate cognitive effort for language production, reflected in looking time.

Most studies have employed an eye-tracking paradigm to study the production of simple phrases. Albeit few, some studies have examined eye movements during sentence- or connected speech-level production. Spieler and Griffin (2006) investigated the description of a simple array of objects into simple sentences following a fixed format (“The A and the B are above the C”) for the older and younger adults. Their findings revealed that older adults exhibited longer fixation durations on the picture before initiating the first object (“the A”) compared to those for younger adults. This suggested that older adults required more time to prepare and retrieve the name of the first noun before initiating the sentence. Jang et al. (2021) examined connected speech and eye movements during the description of the ‘Cookie Theft’ picture (Goodglass and Kaplan, 1972). With the goal of classifying individuals with Alzheimer’s disease, mild cognitive impairment, and subjective memory complaints from healthy controls, they analyzed a linguistic variable (e.g., information units) and eye movement measures (e.g., eye fixations). Findings from machine learning experiments showed that the combination of eye movement and language features yielded the most accurate classification performance, highlighting the effectiveness of eye-tracking data in assessing language production skills. Additionally, Holšánová (2008) investigated connected speech in the description of complex, realistic scenes, with a focus on the temporal relationship between eye fixations and verbal descriptions. During the message planning stage, eye fixations tended to shift to the next region to be described, indicating the preparation stage for language production.

Drawing upon these findings, which suggested that eye movements indicate cognitive processing and reveal patterns of pre-fixation while planning language production, the current study aimed to investigate age-related changes in the planning process of connected speech by analyzing eye fixations. Specifically, we focused on pre-speech fixations, those fixations that occur during the planning stage of the utterance. Our approach is guided by the hypothesis that the planning and retrieval of words or information units influence speech onset and timing, drawing from previous research findings (Griffin and Oppenheimer, 2006; Griffin and Spieler, 2006). Building upon the established temporal correlation between eye movements and verbal descriptions of scenes, where fixations on objects typically precede their corresponding naming (Meyer et al., 1998; Griffin and Bock, 2000; Holsanova, 2001; Meyer and Dobel, 2003; Meyer, 2004), we seek to explore whether older speakers engage in an increased pre-speech preparation which would be reflected in their eye movements during the picture description tasks.

This study aimed to examine differences in connected speech production between older and younger adults, focusing on linguistic (informativeness and productivity) and real-time (eye fixation) measures, using two picture types. Participants were presented with a standardized Beach picture and a culturally adapted Han River picture, from which connected speech samples were obtained while eye movements were recorded. The study addressed two primary questions. Firstly, we anticipated that older adults would demonstrate decreased word retrieval abilities in terms of informativeness (CIUs per minute) and productivity (noun and verb count per utterance). We expected the Han River picture to elicit greater differences between the two age groups due to its cultural and linguistic modifications, which include a more diverse array of familiar actions and objects occurring in the culturally symbolic background of the Han River. Secondly, we hypothesized that older adults would exhibit decreased processing efficiency in connected speech production, as evidenced by increased pre-speech fixation count and duration. Additionally, we anticipated that the Han River picture would evoke larger group differences, as it would require more cognitive effort to process due to the greater number of elements to describe compared to the Beach picture.

The research questions are as follows.

1. Are there age-related differences in linguistic measures (CIUs per minute, noun and verb count per utterance) depending on the picture types (traditional vs. modified picture)?
2. Are there age-related differences in real-time eye movement measures (pre-speech fixation count, duration) depending on the picture types (traditional vs. modified picture)?

## 2 Materials and methods

### 2.1 Participants

A total of 25 younger and 25 older adults consented to participate in the study, which was approved by the Institutional Review Board on Human Subjects of Ewha Womans University (No. ewha-202209-0028-01). All participants (a) were native Korean speakers, (b) had a normal or corrected-to-normal vision, (c) reported no history of neurological or psychiatric diseases based on a health screening questionnaire (Christensen et al., 1992), and (d) showed a normal range of performance in Korean Mini-Mental State Examination (K-MMSE; Kang, 2006; age- and education-adjusted scores >16th percentile). Additionally, the older adults presented within the normal range in (a) a Short version of the Geriatric Depression Scale (S-GDS; Jung et al., 1997; scored below 7) and (b) the Seoul Verbal Learning Test (SVLT; Kang et al., 2012; age- and education-adjusted scores >16th percentile). The K-MMSE and SVLT are parts of the standardized neuropsychological assessments that are part of the Seoul Neuropsychological Screening Battery-II (SNSB-II; Kang et al., 2012). These tests were conducted to ensure that all participants were cognitively healthy.

**Figure 1:**
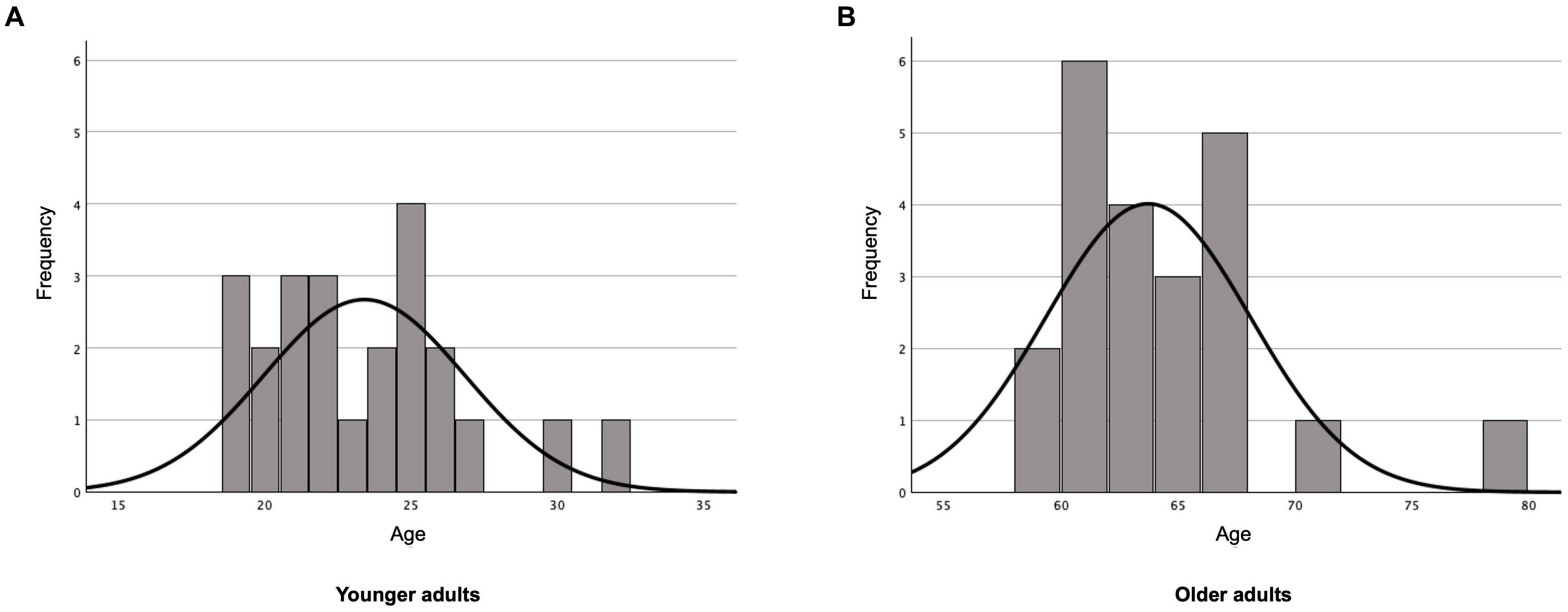
Histograms of participants by age. **(A)** Younger adults, **(B)** older adults.

The quality of eye movement recordings was visually inspected in Eyelink Data Viewer 4.2.1 (SR Research Ltd., 2020b). One younger and three older participants were excluded from the final analysis due to the errors caused by eyelashes or eye makeup, excessive eye blinking, or head movements during the task. The final analysis included 24 younger (Mean age = 23.54 years, SD = 3.44, range = 19–32 years, 15 women) and 22 older adults (Mean age = 63.73 years, SD = 4.38, range = 59–78 years, 13 women). There were no significant differences in years of education (*t*_30.036_ = 0.277, *p* > 0.05) between the younger adults (Mean years of education = 13.82 years, SD = 3.065) and the older adults (Mean years of education = 14.25 years, SD = 1.511) (Figure 1 and Table 1).

**Table 1:** Descriptive information for participants.

Characteristic	Younger adults^a^	Older adults^b^	Test statistics	*p*-value
Gender (male:female)	9:15	9:13	–	–
Age (yrs)	23.54 (3.44) 19–32	63.73 (4.38) 59–78	–	–
K-MMSE	29.75 (0.44) 29–30	29.09 (0.87) 27–30	–	–
Years of education	14.25 (1.51) 12–18	13.82 (3.07) 9–18	0.277	>0.05

Values are presented as means (SD). ^a^*n* = 24, ^b^*n* = 22. K-MMSE = Korean-Mini Mental State Examination (Kang, 2006).

### 2.2 Eye-tracking

During the picture description task, each participant’s eye movements were recorded using an Eyelink Portable Duo (SR Research Ltd.) at a sampling rate of 1,000 Hz. The participants were seated approximately 650 mm from the top of the monitor, 690 mm from the bottom of the monitor, and 450 mm from the eye-tracker camera. To ensure a stable head position, participants placed their foreheads and chins against the rest while their monocular eye movements were recorded. The experiment was programmed using Experiment Builder 2.3.1 (SR Research Ltd., 2020a), and the picture stimuli were presented on a 550 mm × 300 mm monitor screen with a resolution of 1680 × 1050 pixels. Each participant’s speech during the task was recorded via a sound card (model M-Audio Air 192 I 4) using a condenser microphone (model BM800).

Each participant was tested individually in a soundproof room. Before the task began, a practice trial was conducted using a standardized picture from the Screening Test for Aphasia and Neurogenic-communication Disorders (STAND; Kim et al., 2009). During the practice trial, participants were instructed to verbally describe everything happening in the picture using complete sentences. The picture depicted a crosswalk with various individuals on the crosswalk, and participants were required to provide detailed descriptions about who was doing what activities at what place while the picture was displayed on the monitor. Additionally, participants were prompted to indicate when they had finished by saying, “I’m done.” The practice session ended when each participant provided his/her explanations for all the elements in the picture and signaled that they had completed the task.

**Figure 2:**
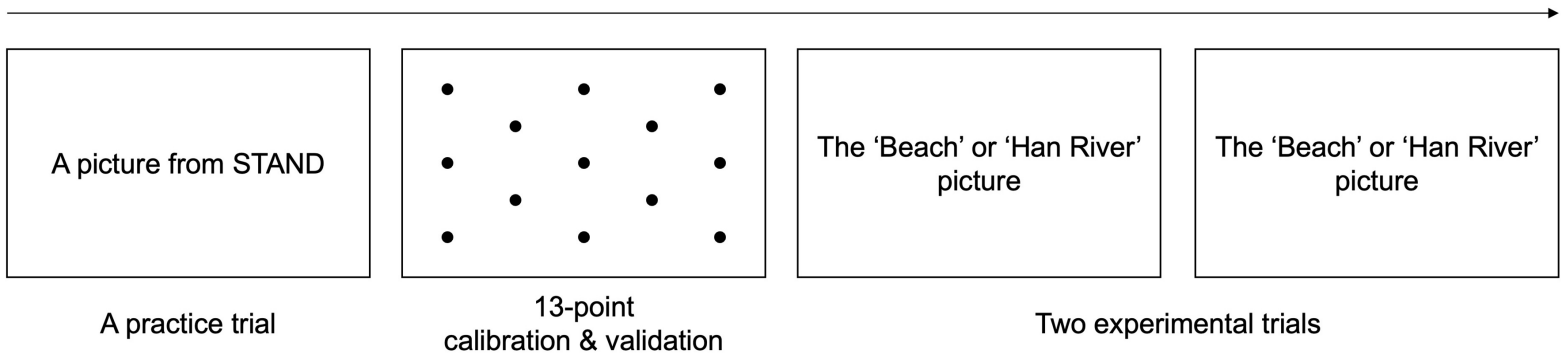
Experimental procedures.

After the practice session, each participant was informed about the calibration and validation process before the main task of describing the Beach and the Han River, in the same manner as the practice session. The 13-point calibration and validation process was conducted in order to ensure accurate eye tracking. After the calibration and validation with an average error of <0.5° and a maximum error of <1.0°, the computer displayed either the Beach or Han River picture and began recording data. The participants were instructed to describe the pictures at their own pace without a time limit. The order of picture presentation was randomized to prevent any bias. No additional instructions were provided once the participants began the task of describing. When each participant completed one picture and indicated to have done so, he/she was provided with the other picture to describe. The experimental procedures are illustrated in Figure 2. Each participant’s eye movement recordings were then analyzed in EyeLink Data Viewer 4.2.1 (SR Research Ltd., 2020b). The fixation counts and durations of eye movements within Areas of Interest (AOIs) were extracted and documented in a Microsoft Excel file.

### 2.3 Outcome variables

#### 2.3.1 Linguistic measures

The linguistic outcome measures for this study included the number of Correct Information Units (CIUs; Nicholas and Brookshire, 1993) per minute and the counts of nouns and verbs per utterance.

##### 2.3.1.1 Transcriptions and utterances

The connected speech samples obtained from picture description tasks were transcribed verbatim and then segmented into utterances based on the criteria established in the previous studies on connected speech analysis in Korean (Kim et al., 1998; Lee and Kim, 2001). An utterance was defined as a sentence or a shorter unit of language. Thus, each utterance in this study typically was counted for a sentence or phrase related to a specific Area of Interest (AOI).

The segmentation rules for utterances (Kim et al., 1998; Lee and Kim, 2001) are outlined as follows. First, an utterance is segmented at the point of a sentence-ending particle (e.g., ‘*-ta*’). If a sentence-ending particle is present but followed by contextually connected words, those words are included in the same utterance. Second, if a conjunction (e.g., “and”) appears, the utterance is segmented from the conjunction to the sentence-ending particle. Third, an utterance is segmented if a pause of more than 2 seconds occurs between words. Finally, if speech continues with a conjunctive suffix (e.g., ‘*-ko*’), segmentation happens at the junction of a significant change in intonation or a pause exceeding 2 seconds. Without such changes or pauses, the utterance extends to the second conjunctive suffix.

##### 2.3.1.2 CIUs per minute

The number of words and CIUs were counted according to the rules of Nicholas and Brookshire (1993). The words in the collected data were initially identified based on the detailed set of rules, adhering to the comprehensive guidelines outlined by Nicholas and Brookshire. The inclusion criterion stipulated that words must be “intelligible in context but did not have to be accurate, relevant, or informative relative to the eliciting stimulus” (Nicholas and Brookshire, 1993, p. 340). Subsequently, among the collected words, those that are “accurate, relevant, and informative relative to the eliciting stimulus” (Nicholas and Brookshire, 1993, p. 340) were designated as CIUs. A CIU corresponded to a single word, and only the words initially counted in the word count were counted for the CIUs (see Nicholas and Brookshire, 1993, for further elaboration). The resulting CIU count was then normalized by the duration of the picture description task in minutes, yielding the measure of CIUs per minute.

##### 2.3.1.3 The number of nouns and verbs

Both nouns and verbs were tallied in terms of ‘type’ or ‘token.’ The number of nouns-token, nouns-type, verbs-token, and verbs-type were counted from the transcriptions of the connected speech data. These measures were then normalized by the number of utterances to mitigate the influence of the total number of utterances on the number of words. The method of tallying the number of nouns and verbs was adapted from Sung et al. (2016). The noun counts included common nouns, proper nouns, and pronouns. The pronouns were counted as nouns for two primary reasons: firstly, pronouns serve a similar grammatical function to content nouns. In Korean grammar, content nouns and pronouns are both frequently marked by case markers to signal grammatical roles in sentences. Secondly, pronouns have specific referents and function similarly to content nouns by referring to specific contents depicted in the pictures (Sung et al., 2016).

In the counts of verbs, both regular verbs and auxiliary verbs were included to capture verbs involved in serial verb construction, which is one of the characteristics of Korean verb usage. Auxiliary verbs are also expressed as distinct verbs with semantic significance in the Korean language (Lee, 1976; Sohn, 1996) and are integrated with the preceding main verbs, functioning as independent syntactic elements. They convey information regarding not only tense and aspect but also psychological attitudes (Park, 2005; Hwang et al., 2009). For example, the auxiliary verb *‘ju-da’* (‘give’) is appended to the main verb *‘nol-da’* (‘play’) to form *‘nol-a juda’* (‘play-give’, to allow him/herself a play with someone).

#### 2.3.2 Eye movement measures

##### 2.3.2.1 Areas of interest (AOIs)

Previous studies have utilized the concept of information units to identify key components of a picture and defined corresponding Areas of Interest (AOIs) (e.g., Jang et al., 2021). In this study, AOIs in the pictures were selected to correspond to the expected CIUs. The Han River picture was assigned 12 AOIs, comprised of nine AOIs of people or animals (active agents) and three AOIs of background elements (static objects). Those AOIs encompassed (1) a family eating a lunch box, (2) a girl lying on the lawn, (3) a girl listening to music, (4) a boy kicking a ball, (5) a boy throwing a ball with his dog, (6) three friends taking a picture, (7) a man running along the track, (8) a man drinking beverage, (9) two bikers, (10) a yacht, (11) buildings, and (12) a tree. Twelve AOIs were also defined for the Beach picture which consisted of six AOIs depicting people or animals (active agents) and six AOIs of background elements (static objects). The AOIs included (1) a family building a sandcastle, (2) a woman reading a book, (3) a guitar, (4) a parasol, (5) a barking dog, (6) a couple walking together, (7) three friends playing volleyball, (8) the sea, (9) a yacht, (10) seagulls, (11) condominiums, and (12) a mountain.

**Figure 3:**
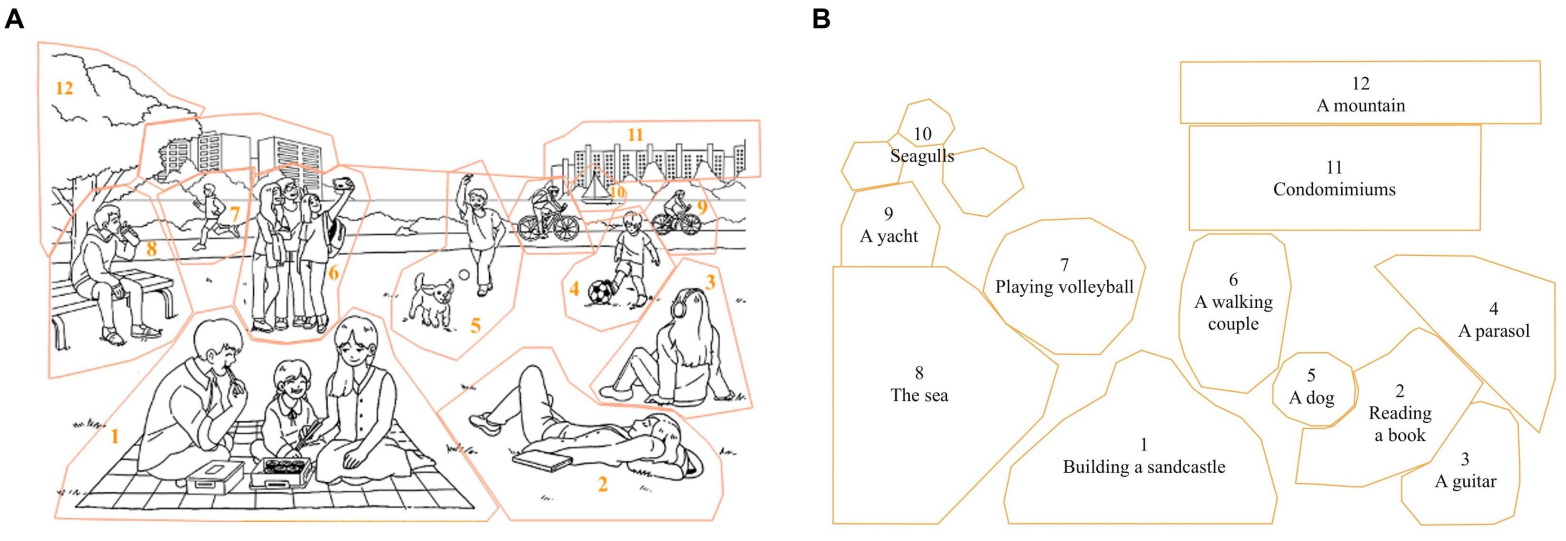
**(A)** Areas of interests (AOIs) of the ‘Han River’ picture. (Patent no. D2022-0004KR; Jeong et al., 2023). **(B)** AOIs of the ‘Beach’ picture (only outlines of AOIs were depicted due to the absence of permission to use copyrighted material).

The AOIs were delineated in a freehand shape using the EyeLink Data Viewer 4.2.1 (SR Research Ltd., 2020b). Given the picture’s crowded nature, the AOIs were set with a buffer around the individuals and objects. These AOIs were slightly larger than the targets themselves, ensuring that eye fixations recorded in the proximity to the target would still be included in the AOIs. Table 2 and Figure 3 illustrate AOIs in two pictures.

**Table 2:** Areas of interests (AOIs).

AOI number	Han River picture	Beach picture
1	A family eating a lunch box	A family building a sandcastle
2	A girl lying on the lawn	A woman reading a book
3	A girl listening to music	A guitar
4	A boy kicking a ball	A parasol
5	A boy throwing a ball with his dog	A barking dog
6	Three friends taking a picture	A couple walking together
7	A man drinking beverage	Three friends playing volleyball
8	A man running along the track	The sea
9	Two bikers riding bicycles	A yacht
10	A yacht	Seagulls
11	Buildings	Condominiums
12	A tree	A mountain

##### 2.3.2.2 Pre-speech fixation count and duration

The starts and ends of each utterance in the connected speech samples were manually checked in milliseconds using Praat software (Boersma and Weenink, 2022). For the eye fixation measures, pre-speech fixations included fixations on AOIs which occurred right before each participant began his/her AOI-related utterances. It is important to note that only the AOI-related utterances were considered, as those picture elements were expected to be described in the form of CIUs during the picture description task. For example, if an eye fixation lands on an AOI representing a dog, the corresponding utterance should ideally contain the CIU ‘dog’. If the utterance did not specifically contain information about the AOI, no pre-speech fixations were counted. For example, statements of personal thoughts such as “This picture reminds me of my vacation” were excluded from the eye movement measurements since a direct relationship could not be verified between the visually attended region in the picture and the spoken description.

**Figure 4:**
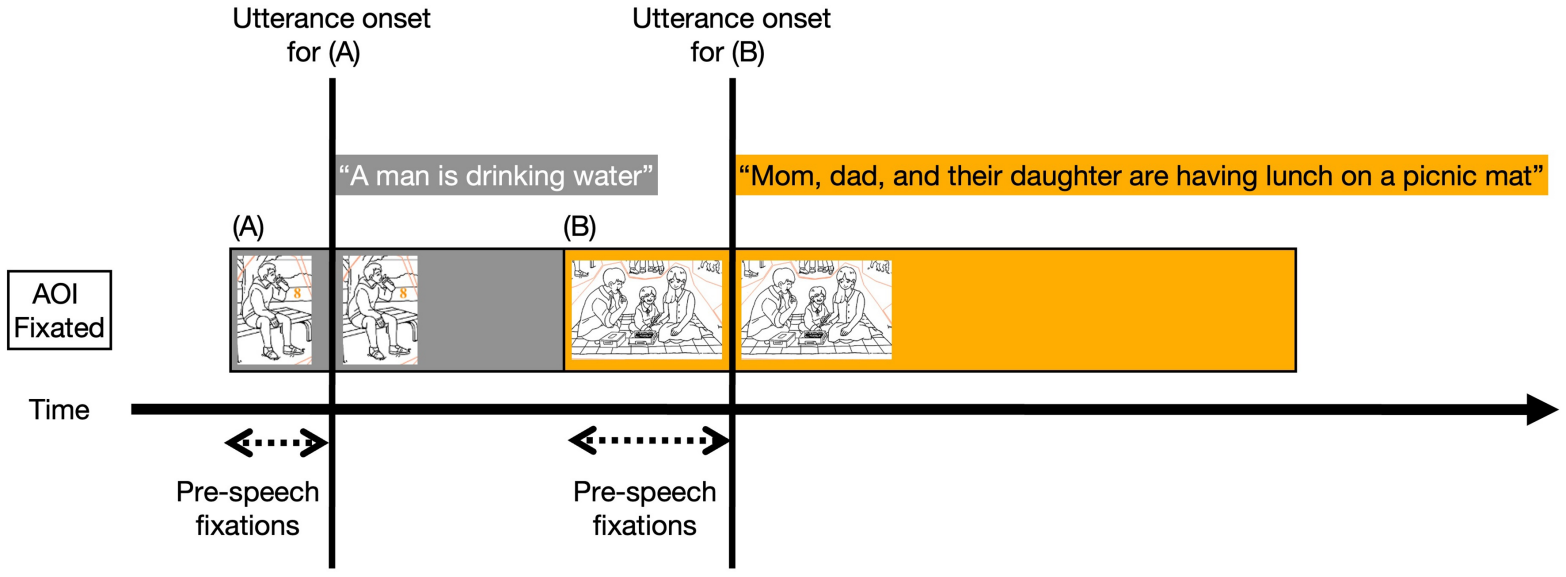
Idealized Illustration of Pre-speech Fixations. The duration of fixations is represented by box size. The figure is adapted from Griffin and Oppenheimer (2006).

To determine pre-speech fixation counts and durations for each utterance, the onset of the previous utterance was set as the starting point and the onset of the current utterance was determined as the endpoint. Within this defined time span, the only eye fixations on the AOI corresponding to the current utterance were counted as pre-speech fixations. A typical pattern of eye fixations in connected speech production is represented in Figure 4. The eyes begin to fixate on the man drinking water shortly before the onset of utterance (A): “A man is drinking water.” As the utterance is about to finish, the eyes shift to the next AOI to be described, which is a family having lunch, and then begin the utterance (B): “Mom, dad, and their daughter….” Thus, the pre-speech fixations for (B) are the fixations on the family picture that occur between the utterance onset for (A) and the onset for (B). These pre-speech fixation counts and fixations for each utterance were then aggregated and divided by the total number of AOI-related utterances.

Studies investigating simple phrase or sentence production have examined eye fixations preceding the target word (e.g., Meyer et al., 1998; Griffin and Bock, 2000; Meyer and Dobel, 2003; Meyer, 2004; Spieler and Griffin, 2006). However, in the context of connected speech, which involves higher-level and more complex language production, eye fixations were reported to correspond to larger linguistic units beyond single words or phrases (Holšánová, 2008). Furthermore, studies indicate that language planning unfolds incrementally in that speakers continuously strategize small and sequential units rather than composing entire sentences at once (Griffin, 2001; Wheeldon et al., 2013). This perspective implies that planning for subsequent utterances may begin while simultaneously expressing the current one. Therefore, in this study, pre-speech fixations were counted as those occurring during the duration of the preceding utterance. Through this investigation, we aimed to uncover age-related changes in the covert cognitive processes involved in planning connected speech production.

### 2.4 Data analysis

Statistical analysis was performed using IBM SPSS Statistics (Version 29.0) (IBM Corp., 2021). A two-way mixed-design (2×2) ANOVA was employed to explore potential interactions between age groups (young vs. older adults) and between the two picture types (‘Han River’ vs. ‘Beach’) using the linguistic (CIUs per minute) and eye-tracking measures (pre-speech fixation count and duration). Additionally, a three-way mixed-design (2×2×2) ANOVA was conducted to investigate noun and verb counts for the variables of age, picture types, and parts of speech (noun vs. verb).

## 3 Results

### 3.1 Linguistic measures

#### 3.1.1 CIUs per minute

**Figure 5:**
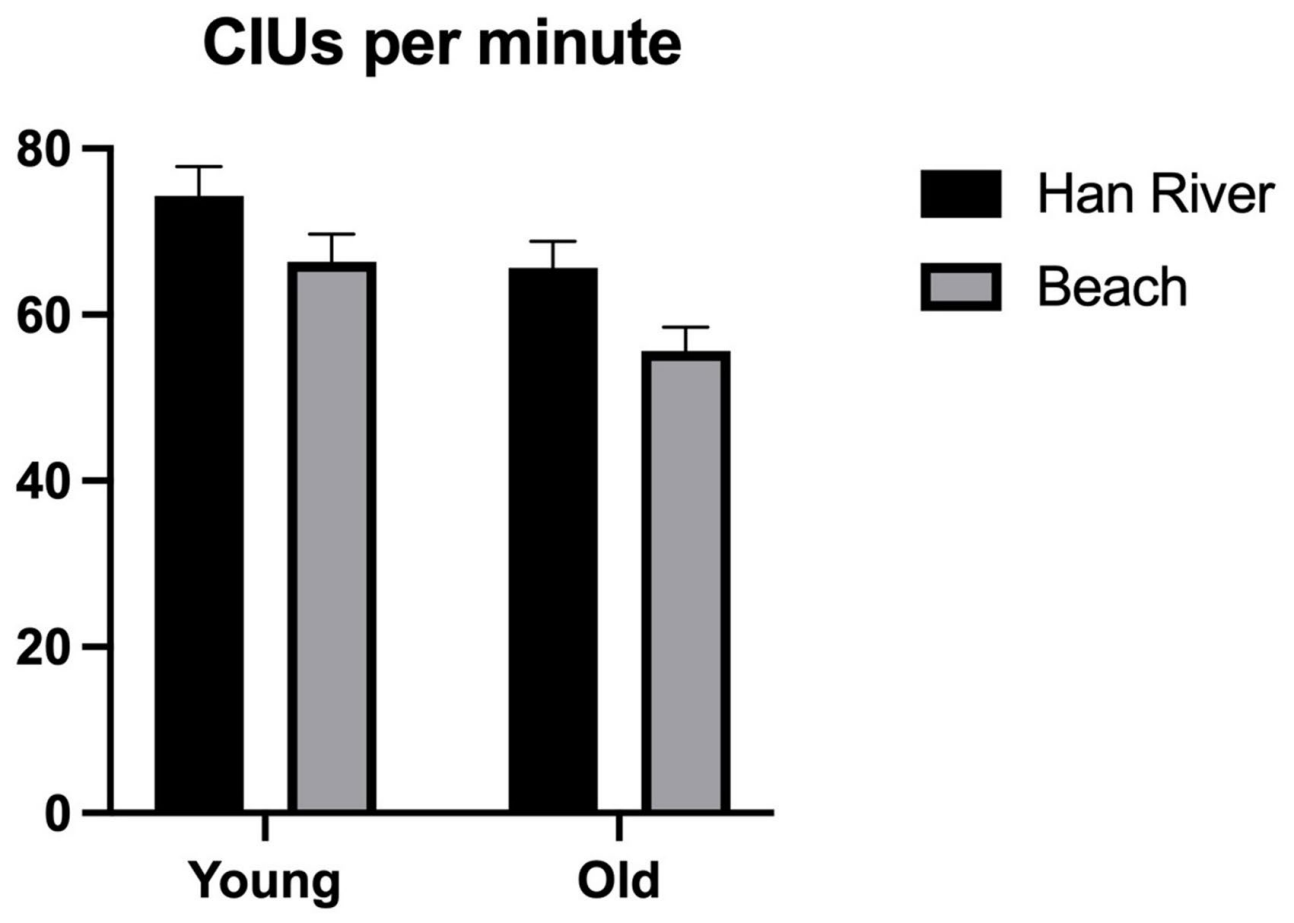
CIUs per minute.

The analysis for CIUs per minute showed a significant main effect for the picture types [*F*_(1,44)_ = 26.812, *p* < 0.001, *η_p_^2^* = 0.379] and group [*F*_(1,44)_ = 5.324, *p* = 0.026, *η_p_^2^* = 0.108]. The Han River picture elicited more CIUs per minute than the Beach picture, and younger adults produced more CIUs than older adults. However, there were no significant interactions between the picture types and group [*F*_(1,44)_ = 0.371, *p* = 0.546, *η_p_^2^* = 0.008]. These results are visualized in Figure 5.

#### 3.1.2 Token analysis (nouns and verbs)

Analysis of the tokens revealed significant main effects for picture types [*F*(1,44) = 31.298, *p* < 0.001, *η_p_^2^* = 0.416], group [*F*(1,44) = 19.624, *p* < 0.001, *η_p_^2^* = 0.308], and parts of speech [*F*(1,44) = 192.764, *p* < 0.001, *η_p_^2^* = 0.814]. The Han River picture elicited significantly more tokens than the Beach picture and the younger adults produced significantly more tokens than older adults. A larger number of nouns were produced compared to verbs.

**Figure 6:**
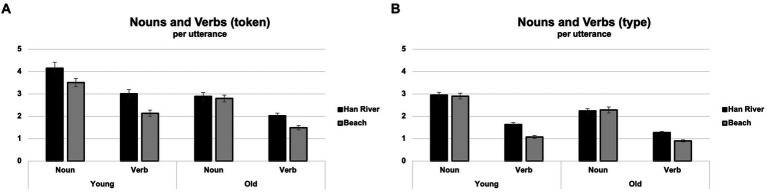
**(A)** Nouns-token, verbs-token, **(B)** nouns-type, verbs-type.

Significant two-way interactions were observed between the picture types and group [*F*(1,44) = 5.385, *p* = 0.025, *η_p_^2^* = 0.109] and between the picture types and parts of speech [*F*(1,44) = 11.017, *p* = 0.002, *η_p_^2^* = 0.200], but not between the parts of speech and group [*F*(1,44) = 0.983, *p* = 0.327, *η_p_^2^* = 0.022]. The younger adults produced significantly more tokens than the older adults and the group difference in tokens was more pronounced for the Han River picture compared to the Beach picture. The Han River picture elicited more tokens than the Beach picture and the difference between the picture types was more prominent in verbs than in nouns. There was no significant three-way interaction among the picture types, groups, and parts of speech [*F*(1,44) = 1.062, *p* = 0.308, *η_p_^2^* = 0.024]. These results are visually represented in Figure 6A.

#### 3.1.3 Type analysis (nouns and verbs)

Analysis of the types showed significant main effects for picture types [*F*(1,44) = 14.842, *p* < 0.001, *η_p_^2^* = 0.252], group [*F*(1,44) = 20.960, *p* < 0.001, *η_p_^2^* = 0.323], and parts of speech [*F*(1,44) = 494.336, *p* < 0.001, *η_p_^2^* = 0.918]. The results showed Han River picture elicited significantly more types than the Beach picture, the younger adults produced significantly more types than older adults, and more nouns were used compared to verbs.

Significant two-way interactions were observed between the groups and parts of speech [*F*(1,44) = 10.429, *p* = 0.002, *η_p_^2^* = 0.192], picture types and parts of speech [*F*(1,44) = 35.807, *p* = <0.001, *η_p_^2^* = 0.449], but not between the picture types and groups [*F*(1,44) = 1.260, *p* = 0.268, *η_p_^2^* = 0.028]. The younger adults produced a significantly higher number of types than the older adults, and the difference between the groups was more pronounced in the nouns than the verbs. The Han River picture elicited more types than the Beach picture, and the difference between pictures was more prominent in the verbs than nouns. No significant three-way interaction was observed amongst the picture types, group, and parts of speech [*F*(1,44) = 0.426, *p* = 0.518, *η_p_^2^* = 0.010]. These results are visually represented in Figure 6B.

### 3.2 Eye movement measures

**Figure 7:**
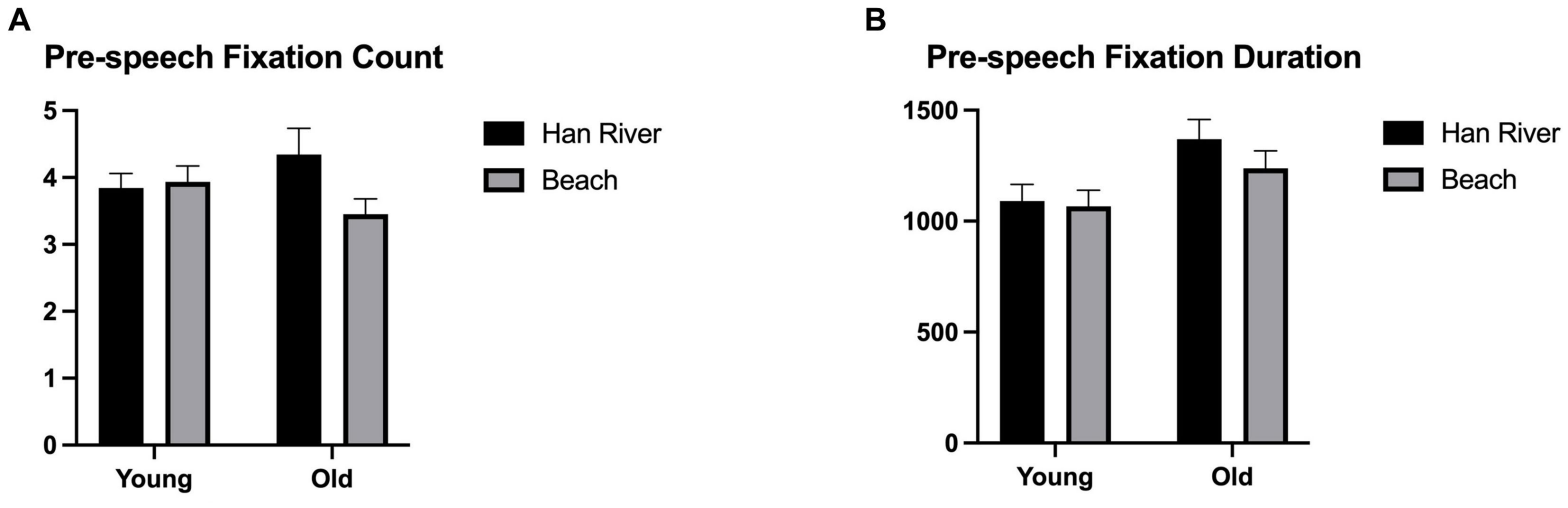
**(A)** Pre-speech fixation count (# counts/# utterances), **(B)** pre-speech fixation duration [durations (ms)/# utterances].

Results on the pre-speech fixation count indicated a significant main effect for the picture types [*F*_(1,44)_ = 4.438, *p* = 0.041, *η_p_^2^* = 0.092] but not for the group [*F*_(1,44)_ = 0.001, *p* = 0.979, *η_p_^2^* = 0.000]. The ‘Han River’ picture elicited a higher pre-speech fixation count for both groups, while there was no statistically significant difference between the groups. A significant interaction between the picture types and group was found [*F*_(1,44)_ = 6.748, *p* = 0.013, *η_p_^2^* = 0.133]. The older group exhibited significantly more fixation counts on the Han River than those on the Beach picture, whereas the younger group did not show significant differences between the two pictures. These outcomes are shown in Figure 7A.

In terms of the pre-speech fixation duration, the main effect was not significant for the picture types [*F*_(1,44)_ = 2.468, *p* = 0.123, *η_p_^2^* = 0.053] but significant for the groups [*F*_(1,44)_ = 5.067, *p* = 0.029, *η_p_^2^* = 0.103]. The older adults showed a longer duration of fixation than that of younger participants. No significant interaction was between the picture types and groups [*F*_(1,44)_ = 1.216, *p* = 0.276, *η_p_^2^* = 0.027]. Figure 7B provides a visual display of these results.

Words per minute (WPM) were analyzed to examine the potential impact of different speech rates of age groups on pre-speech fixations. The results of the independent samples t-tests showed no significant difference in WPM between the younger and older adult groups for either of the ‘Han River’ (*t*_44_ = 1.671, *p* = 0.102) or ‘Beach’ (*t*_44_ = 1.122, *p* = 0.268) pictures. Therefore, any potential differences in pre-speech fixation duration between groups could not be attributed to differences in speech rate.

## 4 Discussion

The purpose of this study was to investigate age-related differences in connected speech produced for a picture description task using two pictures: a standardized picture (‘Beach’) and a culturally and linguistically tailored picture (‘Han River’). The investigation employed linguistic measures (i.e., informativeness and productivity of connected speech) and real-time eye movement measures (eye fixations). The hypothesis was that older adults would demonstrate reduced CIUs per minute and fewer noun and verb counts per utterance, which could indicate age-related declines in informativeness and productivity. Additionally, we explored the possibility of the older adult’s increased pre-speech fixation duration and count during the task, which would indicate greater cognitive processing effort and reduced processing efficiency in producing connected speech. Furthermore, the effects of picture types on the connected speech production were also explored. The hypothesis was that the Han River picture, which was designed with considerations for Korean cultural and linguistic factors, was expected to elicit greater age-related differences than the other picture.

### 4.1 Age-related changes in linguistic measures

Age-related differences were investigated in linguistic measures for the Beach and the Han River picture. We analyzed CIUs per minute (CIUs/min) and the noun and verb types and tokens per utterance (NV types and tokens). Our findings revealed significant group differences in all linguistic measures: the older adults demonstrated a marked decrease in informativeness (CIUs/min), lexical quantity (NV tokens), and lexical diversity (NV types) compared to those of younger adults. Our findings were consistent with previous research in that older adults convey less informational content in connected speech compared to their younger counterparts (e.g., Kemper et al., 2001).

Another age-related difficulty was in verb production in which the older adults showed decreased type and token counts. In our study, older adults tended to convey non-specific verbs, while the younger adults employed specific verbs to vividly describe many actions in the picture. For example, the younger adults might say, “Three seagulls are flying in the sky,” whereas the older adults might simply state that “There are seagulls too.” This non-specific verb usage in older adults may be attributed to the semantic and syntactic requirements in verbs in the Korean language. Given that Korean sentences follow a Subject-Object-Verb (SOV) construction, verb arguments must be activated and structured before the verb is produced in a grammatically correct sentence structure. Consequently, older adults may encounter challenges in verb production due to the increased cognitive load associated with this process. Our findings of reduced verb production are consistent with a previous study in regard to older adults’ challenges in generating heavy verbs compared to light verbs in older adults (Choi et al., 2021). Additionally, difficulties in verb production relative to nouns have been reported in both healthy older adults and individuals with mild cognitive impairment during verbal fluency tasks (Östberg et al., 2005; Stokholm et al., 2013; Clark et al., 2014). Our findings suggest that the age-related decline in verb production observed at the single-word level may extend to connected speech.

Our results revealed that the Han River picture elicited a higher number of CIUs/min and NV types and tokens compared to the Beach picture. This difference could be attributed to the Han River picture portraying more objects and actions, thus facilitating the elicitation of information units and verb production in Korean speakers. Korean is considered a verb-salient language, which makes eliciting a sufficient number of verbs crucial in assessing language production abilities in this population. Furthermore, culturally familiar elements like the setting of the Han River or the daily life activities depicted in the picture may have contributed to eliciting more words. The participants’ prior knowledge and own experiences might have facilitated easier identification and description of the elements and activities in the Han River picture. Our findings support the premise of incorporating cultural and linguistic factors in picture stimuli for language assessments (Evans et al., 2022; Steinberg et al., 2022) in order to effectively evaluate speakers’ language production abilities.

The analysis of group differences between the two picture types revealed that the age-related decline in NV tokens was more pronounced in the Han River picture compared to the Beach picture. This discrepancy seems to pinpoint the decreased productivity in older adults, especially for the Han River picture, where detailed descriptions were required for diverse people, objects, and actions. These findings support that the Han River picture would be sensitive to age-related distinctions, particularly in terms of productivity measures. Even with richer contents in the Han River picture, older adults did not elaborate as extensively as their younger counterparts.

### 4.2 Age-related changes in eye movement measures

Age-related differences were explored in real-time eye movement measures for the picture types. Two measures, the count and duration of pre-speech fixation, were employed to investigate younger and older adults’ cognitive processing efficiency during the planning stage of connected speech production. These measures included eye fixations preceding each utterance rather than encompassing all fixation values throughout the picture description task. This was to understand the cognitive efforts specific to planning utterances within continuous connected speech production.

The analysis of pre-speech fixation duration revealed significant age-related differences. The older individuals exhibited longer pre-speech fixation duration, indicating increased cognitive efforts required for encoding and planning the utterances about those fixated AOIs. The effort hypothesis states that eye fixations reflect attention and mental effort (cf. Griffin, 2004). Longer and more frequent fixations would indicate greater cognitive efforts required for processing information at the AOIs, while shorter and a skipping manner of fixations would imply decreased cognitive processing (Rayner, 2009; Conklin et al., 2018). Additionally, longer pre-speech fixation durations could be interpreted as indicative of slowed processing. The theory of general cognitive slowing (Salthouse, 1996) posits an overall deceleration in cognitive processes among older adults, impacting the efficiency of language processes. In this context, the duration would reflect the time course of language planning processes and would imply reduced efficiency due to the prolonged time required for planning and initiating utterances. Our results are in line with the findings of the previous research. Spieler and Griffin (2006) reported that older participants showed longer fixation durations on the first noun when generating simple sentences compared to younger adults. Verhaegen and Poncelet (2013) noted that older adults showed increased latencies in single-word retrieval in picture naming tasks. Considering the age-related decline in CIUs per minute and noun and verb count along with the longer pre-speech fixation duration, older adults required more time to prepare their utterances than young adults while still displaying a lower level of content measures, such as CIUs per minute and the number of nouns and verbs produced.

The analysis of pre-speech fixation count revealed a significant difference between the picture types; the Han River picture induced greater fixation counts. A significant interaction between the groups and picture types was observed. While the older adults showed significantly greater pre-speech fixation counts in the Han River picture, the younger adults showed no significant difference between the two pictures. This suggests that, unlike younger adults, the older adults might have responded to the increased complexity of the Han River picture. The Han River picture included more referents to describe compared to the less referents in the Beach picture. This finding could be interpreted as the increased cognitive demand may have tapped into the older adults’ experiences in the more complex picture description task. It has been studied that visually complex (e.g., highly cluttered) scenes tend to elicit more words in verbal descriptions (Clarke et al., 2013). The increased visual complexity and demands to produce more words may have posed additional cognitive challenges for older adults. Notably, this discrepancy was not observed among younger adults. Older adults seem to be more susceptible to visual complexity during picture descriptions, showing declined efficiency in language planning in the more complex picture description task. Our findings suggest that the Han River picture appears to demonstrate its sensitivity in distinguishing between age groups compared to the Beach picture. The modified picture effectively addresses some inherent limitations of the traditional image while effectively eliciting information units, particularly verbs.

Both pre-speech fixation duration and counts revealed patterns of inefficient cognitive processing for planning connected speech production in older adults. Previous literature has also employed eye movements as a real-time index in sentence production and reported decreased efficiency in cognitive processing for sentence production in people with aphasia. Several studies have shown that as the complexity of sentence production increases, people with aphasia display different fixation patterns compared to normal adults. Shin and Sung (2020) interpreted these patterns as a sign of difficulty in allocating thematic roles, leading to more errors in producing passive sentences. Additionally, Thompson et al. (2007) found that eye fixation patterns indicated greater processing difficulty when producing sentences with verbs that have complex argument structures in people with aphasia. Our study supports these findings and further extends the discussion on real-time indicators of challenges in language production.

### 4.3 Limitations and future implications

A limitation and future implications of this study are as follows. Firstly, more comprehensive measures for the exploration of language and speech fluency could offer a deeper understanding of connected speech production. Future research endeavors might consider incorporating diverse metrics, such as grammaticality and syntactic complexity, in order to examine language production. Another area could involve the aspect of dysfluency, which often signals word retrieval challenges in older adults (Bortfeld et al., 2001; Castro and James, 2013). Beyond the quantitative analysis of linguistic measures employed in this study (i.e., CIUs per minute, noun and verb counts per utterance), investigating speech fluency measures (i.e., fillers, hesitations, and pauses) could offer other insights into critical aspects of older adults’ connected speech production.

Second, mapping the instances of disfluency with eye fixations during language production could deepen our understanding of the cognitive processes involved. Qualitative analysis of eye movements at the moments of speech disfluency could illuminate the interplay between speech disfluency and visual attention during language production. Moreover, although our study aimed to explore the cognitive processing involved in connected speech production, the specific cognitive demands required in connected speech production remain unspecified. Future research could target various cognitive functions using in-depth assessment to explore potential correlations between specific cognitive functions and pre-speech fixations.

Third, while our study did not primarily investigate social factors as independent variables, previous research has suggested a relationship between older adults’ cognitive and linguistic abilities and various social factors. For instance, social isolation and segregation in older adults’ later years in life have been linked to cognitive declines (Read et al., 2020; Cardona and Andrés, 2023; Ren et al., 2023). Additionally, socioeconomic status (SES) has been associated with cognitive ability in older age. The lower SES has been correlated with lower cognitive ability or cognitive decline (Zahodne et al., 2015; Park et al., 2017; Muhammad et al., 2022; Shi et al., 2023). Future studies could explore the impact of social factors on the connected speech of the aging population.

Also, there is a possibility that social-interactional factors may have influenced the volubility of older adults in our study. While the fixation data suggest slowed processing, it may be plausible that the more limited language output observed in older adults could be influenced by their attitude toward the task and the experimenter. Unfortunately, our analysis did not allow us to disentangle these factors. Addressing the influence of social-interactional factors in future studies could provide valuable insights into age-related differences in language production.

Lastly, although we did not incorporate measures related to eyesight, it is plausible to note that age-related changes in eyesight at the physiological level could potentially influence eye movement measures. Spieler and Griffin (2006) suggested that aging may impact the utilization of extrafoveal information, as there is a decline in extrafoveal sensitivity associated with age. “Extrafoveal” refers to the area of vision outside the central fovea of the retina, which is responsible for sharp central vision. The extrafoveal region encompasses the surrounding peripheral visual field. Extrafoveal visual information may be utilized to gather information about an object while individuals are moving their eyes to begin identifying it. Typically, directing attention to an object occurs approximately 200 ms faster than moving the eyes to the object’s location. Younger adults may preprocess objects before fixation more efficiently than their older counterparts, potentially resulting in shorter fixation durations or fewer fixations due to their relatively physiologically sensitive and faster abilities to explore extrafoveal information (Spieler and Griffin, 2006). In future research, it would be beneficial to investigate how age affects peripheral vision. If this influence is significant, efforts could be made to statistically control for oculomotor changes when analyzing eye movement measures for language processing.

In conclusion, the current study examined the impact of aging on connected speech and eye movement measures during the picture description tasks, utilizing both standardized and modified pictures that incorporated Korean cultural and linguistic factors. It was demonstrated that older adults produced fewer CIUs/min and NV types and tokens per utterance, indicating reduced informativeness and productivity during connected speech production. The eye movement measures also revealed that older participants experienced cognitive challenges, as evidenced by the longer durations of pre-speech fixation on both of the picture types and more pre-speech fixation counts on the modified picture. These findings highlight the need for further investigation into age-related changes in connected speech production in both linguistic and cognitive processes.

## Data availability statement

The datasets presented in this article are not readily available because of approved ethical conditions. Further inquiries can be directed to the corresponding author JES, jeesung@ewha.ac.kr.

## Ethics statement

The studies involving humans were approved by Institutional Review Board on Human Subjects of Ewha Womans University. The studies were conducted in accordance with the local legislation and institutional requirements. The participants provided their written informed consent to participate in this study.

## Author contributions

HL: Conceptualization, Methodology, Writing – review & editing, Data curation, Formal analysis, Investigation, Software, Visualization, Writing – original draft, Project administration. YC: Resources, Writing – review & editing. JES: Conceptualization, Funding acquisition, Methodology, Supervision, Writing – review & editing, Validation.

## References

[ref1] Ash S. Grossman M. (2015). “Why study connected speech production” in Cognitive neuroscience of natural language use. ed. Willems R. M. (Cambridge, U.K.: Cambridge University Press).

[ref2] Baik S. K. Kim E. J. (2012). Han River renaissance project in semiotics of space. In ruc.udc.es. Universidade da Coruña. Available at: https://ruc.udc.es/dspace/handle/2183/13402

[ref3] Berube S. Nonnemacher J. Demsky C. Glenn S. Saxena S. Wright A.. (2019). Stealing cookies in the twenty-first century: measures of spoken narrative in healthy versus speakers with aphasia. Am. J. Speech Lang. Pathol. 28, 321–329. doi: 10.1044/2018_AJSLP-17-0131 30242341 PMC6437702

[ref4] Boersma P. Weenink D. (2022). Praat: doing phonetics by computer version 6.3.01 [computer program]. Available at: http://www.praat.org/

[ref5] Bortfeld H. Leon S. D. Bloom J. E. Schober M. F. Brennan S. E. (2001). Disfluency rates in conversation: effects of age, relationship, topic, role, and gender. Lang. Speech 44, 123–147. doi: 10.1177/00238309010440020101 11575901

[ref6] Boschi V. Catricalà E. Consonni M. Chesi C. Moro A. Cappa S. F. (2017). Connected speech in neurodegenerative language disorders: a review. Front. Psychol. 8:269. doi: 10.3389/fpsyg.2017.00269 28321196 PMC5337522

[ref7] Boucher J. Slegers A. Brambati S. M. (2019). Cross-sectional analysis of picture descriptions of healthy young and older adults. Neuropsychol. Clin. Appl. 3, 132–145. doi: 10.46278/j.ncacn.20190714

[ref8] Brookshire R. H. Mcneil M. R. (2014). Introduction to neurogenic communication disorders — E-book. St. Louis: Mosby.

[ref9] Capilouto G. J. Wright H. H. Maddy K. M. (2015). Microlinguistic processes that contribute to the ability to relay main events: influence of age. Aging Neuropsychol. Cognit. 23, 445–463. doi: 10.1080/13825585.2015.1118006 PMC4939436 26653413

[ref10] Capilouto G. Wright H. H. Wagovich S. A. (2005). CIU and main event analyses of the structured discourse of older and younger adults. J. Commun. Disord. 38, 431–444. doi: 10.1016/j.jcomdis.2005.03.005 16199238

[ref11] Cardona M. Andrés P. (2023). Are social isolation and loneliness associated with cognitive decline in ageing? Front. Aging Neurosci. 15:1075563. doi: 10.3389/fnagi.2023.1075563 36909946 PMC9995915

[ref12] Castro N. James L. E. (2013). Differences between young and older adults’ spoken language production in descriptions of negative versus neutral pictures. Aging Neuropsychol. Cognit. 21, 222–238. doi: 10.1080/13825585.2013.804902 23799772

[ref13] Chenery H. J. Murdoch B. E. (1994). The production of narrative discourse in response to animations in persons with dementia of the Alzheimer’s type: preliminary findings. Aphasiology 8, 159–171. doi: 10.1080/02687039408248648

[ref14] Cho S. Nevler N. Shellikeri S. Parjane N. Irwin D. J. Ryant N.. (2021). Lexical and acoustic characteristics of young and older healthy adults. J. Speech Lang. Hear. Res. 64, 302–314. doi: 10.1044/2020_jslhr-19-00384 33439761 PMC8632482

[ref15] Choi H. (2020). Word-finding Behaviors of discourse production task in healthy elderly adults. Audiol. Speech Res. 16, 347–355. doi: 10.21848/asr.200064

[ref16] Choi S. Jo E. Sung J. E. (2021). Aging effects on the verb fluency measures using the semantic weight-based analysis. Available at: https://easychair.org/publications/preprint/4HXN

[ref17] Christensen K. J. Moye J. Armson R. R. Kern T. M. (1992). Health screening and random recruitment for cognitive aging research. Psychol. Aging 7, 204–208. doi: 10.1037//0882-7974.7.2.204 1610509 PMC4878448

[ref18] Clark D. G. Wadley V. G. Kapur P. DeRamus T. P. Singletary B. Nicholas A. P.. (2014). Lexical factors and cerebral regions influencing verbal fluency performance in MCI. Neuropsychologia 54, 98–111. doi: 10.1016/j.neuropsychologia.2013.12.010 24384308

[ref19] Clarke A. D. F. Elsner M. Rohde H. (2013). Where’s Wally: the influence of visual salience on referring expression generation. Front. Psychol. 4:329. doi: 10.3389/fpsyg.2013.00329 23785344 PMC3684789

[ref20] Collette F. Schmidt C. Scherrer C. Adam S. Salmon E. (2009). Specificity of inhibitory deficits in normal aging and Alzheimer’s disease. Neurobiol. Aging 30, 875–889. doi: 10.1016/j.neurobiolaging.2007.09.007 18029058

[ref21] Conklin K. Pellicer-Sánchez A. Carrol G. (2018). Eye-tracking. Cambridge, U.K.: Cambridge University Press.

[ref22] Connor L. T. Spiro A. Obler L. K. Albert M. L. (2004). Change in object naming ability during adulthood. J. Gerontol. Ser. B Psychol. Sci. Soc. Sci. 59, P203–P209. doi: 10.1093/geronb/59.5.p203 15358792

[ref23] Cooper P. V. (1990). Discourse production and Normal aging: performance on Oral picture description tasks. J. Gerontol. 45, P210–P214. doi: 10.1093/geronj/45.5.p210 2394918

[ref24] Cummings L. (2019). Describing the cookie theft picture. Pragmat. Soc. 10, 153–176. doi: 10.1075/ps.17011.cum

[ref25] Drummond C. Coutinho G. Fonseca R. P. AssunÃ§Ã£o N. Teldeschi A. de Oliveira-Souza R.. (2015). Deficits in narrative discourse elicited by visual stimuli are already present in patients with mild cognitive impairment. Front. Aging Neurosci. 7:96. doi: 10.3389/fnagi.2015.00096 26074814 PMC4446997

[ref26] Evans E. Coley S. L. Gooding D. C. Norris N. Ramsey C. M. Green-Harris G.. (2022). Preliminary assessment of connected speech and language as marker for cognitive change in late middle-aged Black/African American adults at risk for Alzheimer’s disease. Aphasiology 36, 982–1005. doi: 10.1080/02687038.2021.1931801 36016839 PMC9398189

[ref27] Filiou R.-P. Bier N. Slegers A. Houzé B. Belchior P. Brambati S. M. (2019). Connected speech assessment in the early detection of Alzheimer’s disease and mild cognitive impairment: a scoping review. Aphasiology 34, 723–755. doi: 10.1080/02687038.2019.1608502

[ref28] Goodglass H. Kaplan E. (1972). The assessment of aphasia and related disorders. Philadelphia: Lea & Febiger.

[ref29] Griffin Z. M. (2001). Gaze durations during speech reflect word selection and phonological encoding. Cognition 82, B1–B14. doi: 10.1016/s0010-0277(01)00138-x 11672707 PMC5130081

[ref30] Griffin Z. (2004). “Why look? Reasons for eye movements related to language production” in The interface of language, vision, and action: Eye movements and the visual world. eds. Henderson J. M. Ferreira F. (New York: Psychology Press), 213–247.

[ref31] Griffin Z. M. Bock K. (2000). What the eyes say about speaking. Psychol. Sci. 11, 274–279. doi: 10.1111/1467-9280.00255 11273384 PMC5536117

[ref32] Griffin Z. M. Oppenheimer D. M. (2006). Speakers gaze at objects while preparing intentionally inaccurate labels for them. J. Exp. Psychol. Learn. Mem. Cogn. 32, 943–948. doi: 10.1037/0278-7393.32.4.943 16822160 PMC10859217

[ref33] Griffin Z. M. Spieler D. H. (2006). Observing the what and when of language production for different age groups by monitoring speakers’ eye movements. Brain Lang. 99, 272–288. doi: 10.1016/j.bandl.2005.08.003 16290041 PMC5204451

[ref34] Ha J. W. Jung Y. H. Sim H. S. (2009). The functional characteristics of fillers in the utterances of dementia of Alzheimer’s type, questionable dementia, and Normal elders. Korean J.f Commun. Disord. 14, 514–530.

[ref35] Hameister I. Nickels L. (2018). The cat in the tree – using picture descriptions to inform our understanding of conceptualisation in aphasia. Lang. Cogn. Neurosci. 33, 1296–1314. doi: 10.1080/23273798.2018.1497801

[ref36] Heller R. B. Dobbs A. R. (1993). Age differences in word finding in discourse and non-discourse situations. Psychol. Aging 8, 443–450. doi: 10.1037/0882-7974.8.3.443 8216965

[ref37] Henderson J. M. Ferreira F. (2004). “Scene perception for psycholinguists” in The Interface of language, vision, and action: Eye movements and the visual world. eds. Henderson J. M. Ferreira F. (London, U.K.: Psychology Press).

[ref38] Holsanova J. (2001). Picture viewing and picture description: Two windows on the mind [doctoral dissertation].

[ref39] Holšánová J. (2008). Discourse, vision, and cognition. Philadelphia: John Benjamins Pub.

[ref40] Hwang Y. Yi H. Nam K. (2009). The processing and representation of Korean auxiliary verbs in the mental lexicon. Commun. Sci Disord 14, 173–182.

[ref41] IBM Corp. (2021). SPSS statistics for Macintosh (version 29.0) [computer software]. Armonk, NY.

[ref42] James L. E. Burke D. M. Austin A. Hulme E. (1998). Production and perception of “verbosity” in younger and older adults. Psychol. Aging 13, 355–367. doi: 10.1037/0882-7974.13.3.355 9793112

[ref43] Jang H. Soroski T. Rizzo M. Barral O. Harisinghani A. Newton-Mason S.. (2021). Classification of Alzheimer’s disease leveraging multi-task machine learning analysis of speech and eye-movement data. Front. Hum. Neurosci. 15:716670. doi: 10.3389/fnhum.2021.716670 34616282 PMC8488259

[ref44] Jeon Y. M. Kim W. S. (2015). Characteristics of the discourse according to each age group and task type. J. Rehabil. Res. 19, 297–320. doi: 10.16884/jrr.2015.19.1.297

[ref45] Jeong I. Han J. Choi J. Choi Y. Choi S. Sung J. E. (2023). Improving picture description tasks for Korean seniors. J. Digit. Contents Soc. 24, 2767–2775. doi: 10.9728/dcs.2023.24.11.2767

[ref46] Jung I. Kwak D. Joe S. Lee H. (1997). A study of standardization of Korean form of geriatric depression scale (KGDS). J. Korean Geriatr. Psychiatry 1, 61–72.

[ref47] Jung S. Lee C. (2018). Deep neural architecture for recovering dropped pronouns in Korean. ETRI J. 40, 257–265. doi: 10.4218/etrij.2017-0085

[ref48] Kang Y. (2006). A normative study of the Korean-mini mental state examination (K-MMSE) in the elderly. Korean J. Psychol. General 25, 1–12.

[ref49] Kang Y. Jang S. Na D. (2012). Seoul neuropsychological screening battery. 2nd Edn. Seoul: Human Brain Research & Consulting Co.

[ref50] Kavé G. Goral M. (2016). Do age-related word retrieval difficulties appear (or disappear) in connected speech? Aging Neuropsychol. Cognit. 24, 508–527. doi: 10.1080/13825585.2016.1226249 PMC6204153 27583986

[ref51] Kemper S. Sumner A. (2001). The structure of verbal abilities in young and older adults. Psychol. Aging 16, 312–322. doi: 10.1037/0882-7974.16.2.312 11405318

[ref52] Kemper S. Thompson M. Marquis J. (2001). Longitudinal change in language production: effects of aging and dementia on grammatical complexity and propositional content. Psychol. Aging 16, 600–614. doi: 10.1037/0882-7974.16.4.600 11766915

[ref53] Kertesz A. (2007). Western aphasia battery-revised. San Antonio: The Psychological Corporation.

[ref54] Kim H. Huh J. Kim D. Kim J. (2009). Screening test for aphasia and neurogenic-communication disorders (STAND). Seoul: Hakji-sa.

[ref55] Kim W. S. Jeon Y. M. Park B. R. Jang E. J. (2015). An analysis of spontaneous speech of the Elderly’s discourse and topic statement characteristics. J. Special Educ. Rehabil. Sci. 54, 449–464. doi: 10.15870/jsers.2015.09.54.3.449

[ref56] Kim J. Kim H. Namkoong K. Kim S. Kim D. (2006). Spontaneous speech traits in patients with Alzheimer’s disease. Korean J. Commun. Disord. 11, 82–98.

[ref57] Kim H. Kwon M. Na D. L. Choi S. S. Lee K. H. Chung C. S. (1998). Decision making in fluency measures of aphasic spontaneous speech. Commun. Sci. Disord. 3, 5–19.

[ref58] Kim H. Na D. (2012). Paradise-Korean version of Western aphasia battery-revised (PK-WAB-R). Seoul: Paradise Welfare Foundation.

[ref59] Kim H. S. Sung J. E. (2022). Production abilities of predicative adjectives in Korean-speaking individuals with aphasia. J. Speech Lang. Hear. Disord. 31, 9–25. doi: 10.15724/jslhd.2022.31.4.009

[ref60] Kwon M. Kim H. Choi S. S. Na D. L. Lee K. H. (1998). A study for analyzing spontaneous speech of Korean adults with CIU scoring system. Commun. Sci. Disord. 3, 35–49.

[ref61] Kwon N. Polinsky M. Kluender R. (2006). Subject preference in Korean. Proceedings of the 25th West Coast Conference on Formal Linguistics.

[ref62] Lee K. D. (1976). Semantic analysis of auxiliary verbs. Munbeop Yeongou 3, 215–235.

[ref63] Lee Y. Kim H. (2001). An utterance analysis of conversations and picture description tasks of Korean adults. Korean J. Commun. Disord. 6, 40–52.

[ref64] Lee S. J. Kim H. Seo S. Kim M. (2009). Patterns of word class production between picture description and narrative tasks in aphasia. Korean J. Commun. Disord. 14, 470–483.

[ref65] MacKenzie C. (2000). Adult spoken discourse: the influences of age and education. Int. J. Lang. Commun. Disord. 35, 269–285. doi: 10.1080/136828200247188 10912255

[ref66] Marini A. (2023). “Cognitive and linguistic characteristics of narrative discourse production in healthy aging” in Discourse analysis in adults with and without communication disorders. eds. Coelho C. Cherney L. Shadden B. (San Diego: Plural Publishing).

[ref67] Martin R. Slevc L. R. (2012). “Memory disorders and impaired language and communication” in Cognition and acquired language disorders: an information processing approach. eds. Peach R. K. Shapiro L. P. (St Louis: Elsevier), 169–194.

[ref68] Meyer A. S. (2004). “The use of eye tracking in studies of sentence generation” in The interface of language, vision, and action: eye movements and the visual world. eds. Henderson J. M. Ferreira F. (New York: Psychology Press), 191–211.

[ref69] Meyer A. S. Dobel C. (2003). “Application of eye tracking in speech production research” in The mind’s eye: cognitive and applied aspects of eye movement research. eds. Hy J. Radach R. Deubel H. (Amsterdam, Netherlands: North Holland), 253–272.

[ref70] Meyer A. S. Sleiderink A. M. Levelt W. J. M. (1998). Viewing and naming objects: eye movements during noun phrase production. Cognition 66, B25–B33. doi: 10.1016/s0010-0277(98)00009-2 9677766

[ref71] Mueller K. D. Hermann B. Mecollari J. Turkstra L. S. (2018). Connected speech and language in mild cognitive impairment and Alzheimer’s disease: a review of picture description tasks. J. Clin. Exp. Neuropsychol. 40, 917–939. doi: 10.1080/13803395.2018.1446513 29669461 PMC6198327

[ref72] Mueller K. D. Koscik R. L. Turkstra L. S. Riedeman S. K. LaRue A. Clark L. R.. (2016). Connected language in late middle-aged adults at risk for Alzheimer’s disease. J. Alzheimers Dis. 54, 1539–1550. doi: 10.3233/jad-160252 27636838 PMC5137196

[ref73] Muhammad T. Sekher T. Srivastava S. (2022). Association of objective and subjective socioeconomic markers with cognitive impairment among older adults: cross-sectional evidence from a developing country. BMJ Open 12:e052501. doi: 10.1136/bmjopen-2021-052501 PMC9394209 35981779

[ref74] Nicholas L. E. Brookshire R. H. (1993). A system for quantifying the Informativeness and efficiency of the connected speech of adults with aphasia. J. Speech Lang. Hear. Res. 36, 338–350. doi: 10.1044/jshr.3602.338, PMID: 8487525

[ref76] Östberg P. Fernaeus S.-E. Hellström Å. Bogdanović N. Wahlund L.-O. (2005). Impaired verb fluency: a sign of mild cognitive impairment. Brain Lang. 95, 273–279. doi: 10.1016/j.bandl.2005.01.010 16246735

[ref78] Park S. (2005). Study on the syntax and semantic of auxiliary verbs in Korean. Seoul: Yeoknak.

[ref79] Park M. H. Smith S. C. Neuburger J. Chrysanthaki T. Hendriks A. A. J. Black N. (2017). Sociodemographic characteristics, cognitive function, and health-related quality of life of patients referred to memory assessment Services in England. Alzheimer Dis. Assoc. Disord. 31, 159–167. doi: 10.1097/wad.0000000000000166 27819844

[ref80] Rayner K. (1998). Eye movements in reading and information processing: 20 years of research. Psychol. Bull. 124, 372–422. doi: 10.1037/0033-2909.124.3.372 9849112

[ref81] Rayner K. (2009). Eye movements and attention in reading, scene perception, and visual search. Q. J. Exp. Psychol. 62, 1457–1506. doi: 10.1080/17470210902816461 19449261

[ref82] Read S. Comas-Herrera A. Grundy E. (2020). Social isolation and memory decline in later-life. J. Gerontol. 75, 367–376. doi: 10.1093/geronb/gbz152 PMC6963696 31781769

[ref83] Ren Y. Savadlou A. Park S. Siska P. Epp J. R. Sargin D. (2023). The impact of loneliness and social isolation on the development of cognitive decline and Alzheimer’s disease. Front. Neuroendocrinol. 69:101061. doi: 10.1016/j.yfrne.2023.101061 36758770

[ref84] Richardson J. D. Dalton S. G. H. (2019). Main concepts for two picture description tasks: an addition to Richardson and Dalton, 2016. Aphasiology 34, 119–136. doi: 10.1080/02687038.2018.1561417 32952259 PMC7500506

[ref85] Saffran E. M. Berndt R. S. Schwartz M. F. (1989). The quantitative analysis of agrammatic production: procedure and data. Brain Lang. 37, 440–479. doi: 10.1016/0093-934x(89)90030-8 2804622

[ref86] Salthouse T. A. (1990). Working memory as a processing resource in cognitive aging. Dev. Rev. 10, 101–124. doi: 10.1016/0273-2297(90)90006-p

[ref87] Salthouse T. A. (1996). The processing-speed theory of adult age differences in cognition. Psychol. Rev. 103, 403–428. doi: 10.1037/0033-295x.103.3.403 8759042

[ref88] Schmitter-Edgecombe M. Vesneski M. Jones D. W. R. (2000). Aging and word-finding: a comparison of spontaneous and constrained naming tests. Arch. Clin. Neuropsychol. 15, 479–493. doi: 10.1093/arclin/15.6.479 14590203

[ref89] Schotter E. (2011). Eye movements as an index of linguistic processing in language production. J. Psychol. Behav. 11, 16–23.

[ref90] Seçkin M. Savaş M. (2023). Picnic, accident or cookies? A systematic approach to guide the selection of the picture definition tasks in linguistic assessment. Arch. Clin. Neuropsychol. 38, 236–246. doi: 10.1093/arclin/acac109 36594105

[ref91] Shi L. Tao L. Chen N. Liang H. (2023). Relationship between socioeconomic status and cognitive ability among Chinese older adults: the moderating role of social support. Int. J. Equity Health 22:70. doi: 10.1186/s12939-023-01887-6 37095501 PMC10124054

[ref92] Shin M. K. Sung J. E. (2020). Syntactic priming effects on active and passive sentence production in persons with aphasia: evidence from an eye-tracking study. Commun. Sci. Disord. 25, 75–91. doi: 10.12963/csd.20684

[ref94] Sohn S. M. D. (1996). Korean auxiliary verb research. Seoul: Hanshin Publishing Co.

[ref95] Spieler D. H. Griffin Z. M. (2006). The influence of age on the time course of word preparation in multiword utterances. Lang. Cogn. Proc. 21, 291–321. doi: 10.1080/01690960400002133

[ref96] SR Research Ltd (2020a). SR research experiment builder 2.3.1 [computer software]. Oakville, Canada: SR Research Ltd.

[ref97] SR Research Ltd (2020b). SR research data viewer 4.2.1 [computer software]. Oakville, Canada: SR Research Ltd.

[ref98] Steinberg A. Lyden P. D. Davis A. P. (2022). Bias in stroke evaluation: rethinking the cookie theft picture. Stroke 53, 2123–2125. doi: 10.1161/strokeaha.121.038515 35514285

[ref99] Stokholm J. Jørgensen K. Vogel A. (2013). Performances on five verbal fluency tests in a healthy, elderly Danish sample. Aging Neuropsychol. Cognit. 20, 22–33. doi: 10.1080/13825585.2012.656576 22352781

[ref100] Sung J. E. DeDe G. Lee S. E. (2016). Cross-linguistic differences in a picture-description task between Korean- and English-speaking individuals with aphasia. Am. J. Speech Lang. Pathol. 25:140. doi: 10.1044/2016_ajslp-15-0140 27997955

[ref101] Thompson C. K. Dickey M. W. Cho S. Lee J. Griffin Z. (2007). Verb argument structure encoding during sentence production in agrammatic aphasic speakers: an eye-tracking study. Brain Lang. 103, 24–26. doi: 10.1016/j.bandl.2007.07.012

[ref102] Vandenborre D. Visch-Brink E. Dun K. Verhoeven J. Mariën P. (2017). Oral and written picture description in individuals with aphasia. Int. J. Lang. Commun. Disord. 53, 294–307. doi: 10.1111/1460-6984.12348 29119700

[ref103] Verhaegen C. Poncelet M. (2013). Changes in naming and semantic abilities with aging from 50 to 90 years. J. Int. Neuropsychol. Soc. 19, 119–126. doi: 10.1017/s1355617712001178 23237304

[ref104] Wheeldon L. Ohlson N. Ashby A. Gator S. (2013). Lexical availability and grammatical encoding scope during spoken sentence production. Q. J. Exp. Psychol. 66, 1653–1673. doi: 10.1080/17470218.2012.754913 23286440

[ref105] Zacks R. T. Hasher L. (1994). “Directed ignoring: inhibitory regulation of working memory” in Inhibitory processes in attention, memory, and language. eds. Dagenbach D. Carr T. H. (New York: Academic Press), 241–264.

[ref106] Zahodne L. B. Stern Y. Manly J. J. (2015). Differing effects of education on cognitive decline in diverse elders with low versus high educational attainment. Neuropsychology 29, 649–657. doi: 10.1037/neu0000141 25222199 PMC4362867

